# An Ishmaelite

**Published:** 1888-06-23

**Authors:** Leslie Keith

**Affiliations:** Author of "The Chilcotes," "Uncle Bob's Niece," "East and West," etc.


### An Ishmaelite.

By Leslie Keith,

Author of "The Chilcotes," "Uncle Bob's Niece," "East and West," etc.

(Continued from p. 186.)

Chapter XI.—Delia on the Defence

A young man who was manifestly of a different social order could not descend upon this little corner of the world without raising some comment and speculation in the breasts of his new neighbours. He gave himself no airs, it is true, but he kept himself to himself, and that is an unpardonable offence in the eyes of certain people. For the matter of that, the Proudies did the same, and a good many feeble puns had been made from time to time turning on the similarity between the family name and nature.

"My gals ain't of that sort that takes up with heverybody. Them as does never comes to no good, in my opinion," said the good-natured mother, confiding in the taciturn assistant while she poured out his tea. The repressive Miss Betsy was for once absent, and Mrs. Proudie prattled on in happy disregard of grammar and pronunciation. "Their father is perticklar, and, though I say it that shouldn't, there's no girls round here that can come near them for looks or beayveour or learning neither. Proudie, 'e was set on their being scholards. There's Barbara—the books that gel 'as read would make your 'air stand on end on'y just to name. When she 'as an 'eadache its nothin' but them books and hideas knockin' about in her pore 'ead."

"Miss Barbara has read a great deal," said Fred, looking up from his cup and speaking with an effort.

"And so 'as Del, too," cried the mother, jealous of her younger darling. "But Del, she's more took up with the 'ouse, and it's just as well, too, for Proudie, 'e says she's not to go down to the shop no more—not since the lib'ery was opened. She's too pretty, she is, to 'ave the men folks staring at 'er and like as not talking nonsense to 'er, 'e says. And it's true, Mr. Eastgate, my Del is as pretty a gel as you'll find anywheres about."

"Yes," said Eastgate, absently, vaguely understanding that an answer was expected of him.

"There's the Miss Greens over the way"—she plaintively indicated the direction with a wave of her hand—"as holds theirselves above my gels because they can play and drawr and speak the French langwidge, so they tell me. Proudie wouldn't let the gels learn none of them things. 'It ain't fine ladies they was borned to be,' 'e says, 'but useful women. You teach 'em to cook as you can cook, Barbara,' 'e says—for I was a famous 'and at pastry and light dishes, you know, Mr. Eastgate, when I was in service with Mr. Arthur's cousins, where Proudie and me kept company, 'and to sew their shirts when they gets 'usbands of their own, and to say their prayers and to read their Bibles and be good gels, and I'll make you a present of the French langwidge and the pianny playing.' And its true enough, Mr. Eastgate, the Miss Greens is light, and too free-spoken to please me. It's all new pattens and ribbins and finery and sweethearts, and such goings on as never was. You'll see them when the libery is opened, they're sure to come if its only to set eyes on you."

"I think I must be going down now," said Fred, getting up and pushing aside his chair to end these confidences.

To see him? To come to whisper and stare at him as—as——

The hot, sensitive blood rushed to his face as he stumbled down the dark stair, and then he remembered the kindness that had kept his story hidden, and he reddened from another kind of shame this time. The Miss Greens, whose father kept a tinsmith's establishment on the other side of the street and tinkered old pots and pans for the neighbouring housewives, had already made themselves heard, if they had not been visible. For a great part of the day the piano pounding, which went by the name of music, came sounding out of the opposite windows. Miss Maria would rush up the scale with a courageous assault that yet somehow never reached the intended height, and would descend again with a series of fortuitous skips and leaps that took no notice of defaulting notes passed over by the way. This came in time to be a species of torture to Fred, who had a sensitive ear and remembered his mother's gentle music. But it was scarcely a relief when Miss Bell succeeded her sister and crashed upon the chords.

There are quite as inadequate performances to be heard in many drawing-rooms, but then, as a rule, unless you happen to be a governess, you are not forced to listen continuously.

Perhaps on the whole the drawbacks of life in Bermondsey lie chiefly in the fact that you cannot get away from them. The houses are so poorly constructed, so close together, so well peopled, that one lives like a bee in a hive, sharing everything, pleasant or unpleasant, in common. Those of us who inhabit comfortable dwellings do not sufficiently value the blessing of room to be sometimes alone. In this age of irritable nerves and tempers, a little wholesome solitude is sometimes our only salvation. If we lived as the poor do, packed like herrings in a barrel, should we be as patient, as forbearing, as tender of each other's peculiarities?

More solitude was perhaps the one thing Fred would have asked. The little street was full of noises all day long. Itinerant vendors—those merchants of the poor—street singers, beggars, the unemployed who lounged hopelessly, the busy who ran to and fro, all these helped the great volume of sound that fell so strangely on ears used to the rigid silence of a prison. Yet Fred was thankful for that portion of peace that fell to him, and for the lot that gave him the silent Barbara for a companion. The family meals were his chief dread, but in course of time he evaded them, for when custom became busier below he kept the shop while Barbara dined, and took his own food in solitude afterwards.

"He's too 'igh and mighty to set down with common folks," cried the jealous little cripple, who was sometimes detained beyond the family tea-hour and never found that any special preparations were made for his comfort.

"He's different from you," said Delia, pointedly, when she fetched the teapot from the hob for this late comer.

"And so he is from you," said the angry little man. "He's a gentleman."

"I'm glad you've found that out," said Delia, clenching her little hand and speaking with passion. "Is that why you taunt him and sting him, because he is a gentleman, and is poor, and is not ashamed to work?"

"Do you think he would hide himself down here if there wasn't a reason for it?" cried Tucker furiously, rent with jealousy. "Work! Ain't there work for the likes of him among gents? If he's poor, why don't that stuck-up cousin of his put his hand in his pocket? He's flush enough. He seemed mighty fond of him, though he went away and left 'im here. If he's making up to you, this one, he's fooling you. I tell you he ain't your sort; you're not a lady. You're on'y Proudie's daughter, and your mother was a servant."

Delia looked the speaker straight in the face with wrath and contempt in her blue eyes and deep blushes of anger on her cheeks. How pretty she looked in her scorn and contempt—oh, how pretty!

"I'm not a lady," she said; "and I'm not ashamed of being what I am; but I'm ashamed of you. Would you think it manly if Mr. Eastgate were to taunt you with your misfortunes; to scorn you because your back's crooked and your legs are not straight? If there's anything in his life that makes it sad—and I know nothing of him and wish to know nothing—is it any concern of yours? He's a gentleman, as you say, and far above you. It's that that you hate him for, because he is high above you, where you can never reach him."

"You—you despise me because I'm a hunchback, Del." He turned upon her with wrath and despair. "Do you think that I've no feelings, that you trample on them so?"

"It is you who trample on other people's feelings," she said coldly. "And you think because you are sickly and weak that you are to be allowed to say anything you like. It's your crooked mind I hate, George Tucker, and your spite and envy and jealousy." She ended her little outburst, walking out of the room with a high-held head.

But when she climbed up to the bed-room she shared with Barbara, and found her sister there, her overwrought mood relaxed, and she burst out sobbing.

"What is it, Del?" cried Barbara, throwing down her hairbrush, and running to her little sister. She took her in her arms and soothed her. "What is it, Del?" she asked; "has Aunt Betsy——"

"No," sobbed Del, "it is only that little viper, George Tucker. I couldn't listen to him and not speak back. Barbara, why does he taunt me with not being a lady? I should be less of one than I am if I joined him in the things he says."

"Says of whom, dear?"

"Of your helper, Barbara; of Mr. Eastgate. Oh, I don't care for Mr. Eastgate. Why should I? He is dull and sombre enough to frighten any girl, but he is different from us; and yet is not ashamed of us, as Tucker says he is; and if he is in any trouble, isn't it wicked to mock him?"

Barbara's clear grey eyes looked thoughtfully past her sister.

"Yes; he is different," she said, and then she smiled. "George Tucker feels the difference, and he doesn't like it. I don't think you need mind what he says about any of us, Del. I daresay it isn't easy to be charitable when you're made different from other people."

"He's so spiteful," sighed Delia; and indeed Mr. Tucker's fierce admiration and hot jealousy made her life rather burdensome. Presently, hiding her face on her sister's shoulder, she whispered—

"Do you think, Barbara—do you think it is true that he has some reason for hiding down here?"

"I don't know, dear," said Barbara staidly. "Perhaps he has had some great trouble in his life. I cannot tell; and even if—if we should have our guesses, Del, we must put them away; we must not talk about them even to each other. It would be like trying to find out a secret that belongs to somebody else."

"We'll leave that to George Tucker," said Del disdainfully. "I wish father hadn't kept him, he has grown up so disagreeable,"

"Perhaps nobody else would have had him."

"That isn't a reason," said Delia solemnly, shaking her pretty head. "Why should we have him because he is disagreeable?" Then suddenly she lifted her face and looked at her sister. "Barbara," she said, "I think you are very good, though you are not a lady, and though you are only Proudie's daughter and your mother was a servant!"

Barbara laughed out wholesomely.

"No; I'm not a lady, I'm a working woman," she said; "and I mustn't waste time chattering here. Go and give poor George some tea, Del, and make your peace with him."

"Indeed, I won't!" cried the young girl stoutly. "Did you think I was crying for repentance? I was only crying for rage, and I am not going to let him see me with my eyes all red. He would be glad if he thought he had hurt me. Barbara," she clutched at her sister's skirts as she was about to leave the room, "tell me something."

"What am I to tell you?"

"Tell me, do you think the other one—Mr. Eastgate's cousin—will desert him, and be ashamed to come back?"

Barbara looked at the flushed, childish face with a new gravity in her own. She took Delia's chin in her hand, and looked deep into the blue eyes. Then she kissed the pretty pouting lips very tenderly. Barbara's caresses were rather rare, and Delia wondered what she had done to merit this one.

"I cannot tell you, Del," she said at last. "He lives in another world to ours, and I daresay he has many friends and pleasures to keep him there and make this place strange and disagreeable to him. If he does forget it would only be natural."

"You speak as if we were wild beasts," said Del with a pout. "You are worse than George Tucker, Barbara."

Barbara smiled. "Don't quarrel with me, Del, or I'll be worse than George Tucker in another sense, and torment you into friendship. Let me go, child; I've lingered too long."

Did Barbara mean to convey a little hint or warning when she said that it would be only natural for Mr. Arthur Eastgate to forget?

"If he comes again he must not see Del," she thought, as she went back to her duties. It was scarcely likely that a young man could see Del often and yet go and forget her. That kiss of ungrudging love and affection was the seal of a silent resolution on Barbara's part; perhaps a vow to save the little one from so much as an innocent pang.

But the best laid schemes "gang aft aglee," and froward circumstances had already frustrated Barbara's design.

She walked sedately into the shop to find Mr. Arthur Eastgate seated there on the counter, looking very bright, handsome, and smiling, and extremely unlike a native of Bermondsey in the correct evening dress which peeped out under his thin overcoat. He jumped up at Barbara's entrance, and held out a very cordial hand.

Barbara took it in an odd bewilderment. Here was the answer to Del's queries, and what became of her resolves?

"Miss Barbara, I came down for the inauguration of the library, and I find that I've invited myself to a private view."

"We don't open till to-morrow; the catalogue isn't quite ready."

She stole a look at Fred, who stood with a curious half-stern, half-sad frown upon his brow.

"You ought not to have come, Arthur," he said in a low voice.

Arthur laughed, looking frankly at Barbara.

"I will make up for coming on the wrong night by coming again on the right one," he said. "As one of the general public, thirsting for information, you can't refuse to admit me. If I bring my penny I may take my choice, mayn't I?"

"You won't like it," said Barbara, earnestly. "The people are rather rough."

"Be content with your private view," said Fred, rousing himself suddenly. "Let us show you the books now, and don't come again."

Arthur's looks sufficiently expressed his reproach, but before he could clothe it in words Fate once more interposed. Hindering circumstance this time took the shape of Mr. Proudie, bustling in from appeasing other people's appetites to give a moment's satisfaction to his own.

"What, is it you, Mr. Arthur?" he said with honest delight. "You've come to see the show? And a fair set-out it is too, thanks to you, sir. Will you honour us with coming upstairs, and taking a bite with us as a family? I never can touch a morsel in there"—he pointed to the dividing partition with a backward thumb—"it's the smells and the steam that does it." The smells had indeed pursued him, and were almost as satisfying as those seventeen odours of Cologne that are said to be "as good as a taste."

It was yet but little after nine o'clock. Arthur had not finished dinner an hour, and yet he accepted with alacrity this invitation to sup in the Proudie family bosom.

What, indeed, was the use of Barbara's resolves?

*(To be continued.)*

